# A Novel Electroporation System for Living Cell Staining and Membrane Dynamics Interrogation

**DOI:** 10.3390/mi11080767

**Published:** 2020-08-11

**Authors:** Yuanjun Zhang, Zishen Yan, Xingyu Xia, Yuan Lin

**Affiliations:** 1 HKU-Shenzhen Institute of Research and Innovation (HKU-SIRI), Guangdong 518057, China; yjzhang1@connect.hku.hk (Y.Z.); yzs531@connect.hku.hk (Z.Y.); u3005822@connect.hku.hk (X.X.); 2 Department of Mechanical Engineering, The University of Hong Kong, Hong Kong SAR, China

**Keywords:** electroporation, membrane resealing, electrotransfection, live cell staining, cell adhesion

## Abstract

A novel electroporation system was developed to introduce transient membrane pores to cells in a spatially and temporally controlled manner, allowing us to achieve fast electrotransfection and live cell staining as well as to systematically interrogate the dynamics of the cell membrane. Specifically, using this platform, we showed that both reversible and irreversible electroporation could be induced in the cell population, with nano-sized membrane pores in the former case being able to self-reseal in ~10 min. In addition, green fluorescent protein(GFP)-vinculin plasmid and 543 phalloidin have been delivered successively into fibroblast cells, which enables us to monitor the distinct roles of vinculin and F-actin in cell adhesion and migration as well as their possible interplay during these processes. Compared to conventional bulk electroporation and staining methods, the new system offers advantages such as low-voltage operation, cellular level manipulation and testing, fast and adjustable transfection/staining and real-time monitoring; the new system therefore could be useful in different biophysical studies in the future.

## 1. Introduction

### 1.1. Electroporation

Under a high-voltage electric field, transient pores can be created on the cell membrane which then changes its conductivity and permeability, a process referred to as electroporation [1]. By adjusting the magnitude of the applied voltage, the number of pulses and the duration of each pulse, one can in principle control the density, size, and lifetime of membrane [2]. Depending on the ability of those pores to reseal, these pores can be classified as reversible or irreversible. The appearance of pores on the membrane allows exogenous molecules, such as drugs, antibodies, DNA, RNA, oligonucleotides, etc., to enter the cell [3] as well as to selectively kill cells. For this reason, electroporation has become a popular tool in gene transfer, drug delivery, and cancer treatment [4]. In particular, different commercial bulk electroporation (BEP) setups have been developed to achieve high throughput in cargo delivery [5]. However, most traditional BEP methods need high voltage (usually of the order of hundreds of volts) for successful electroporation, which could cause cell death and unanticipated risks in operation. On the other hand, BEP devices are bulky and cannot control or monitor the response of individual cells. Therefore, various micro-fabricated chips were designed to conduct electroporation at single-cell level without the use of high voltage. For example, based on a micro-electroporation system, a recent study has shown that the electroporation efficiency of lung tumor cells is closely related to their resistance against commonly used anti-cancer drugs [6,7], indicating the possibility of utilizing electroporation in the classification and prognosis of cancer.

Another important application of electroporation is small particle delivery for drug loading [8], living cell staining or transfection [9], which is crucial for elucidating the biological roles/duties of different cellular components and organelles. Conventional methods of living cell staining rely on the proper characteristics (like reactivity and hydrophilicity) of staining molecules that allow them to pass through the membranes of living cells spontaneously [10]. However, it usually takes hours for small dyes to enter the cell, making it challenging to achieve immediate and real-time staining and monitoring. For cell transfection, many transfection reagents like polyethyleneimine (PEI) and lipofectamine 2000 are highly cytotoxic, causing serious cell viability issues. In this regard, electroporation could serve as an alternative approach to overcome these limitations. Indeed, various attempts have been made in the past few decades to utilize electroporation setups in labeling cells as well as studying different cellular processes. For example, a nanocapillary electrophoretic electrochemical chip was designed to detect the release of neurotransmitters from a single living cell [11]. Recently, the selective electroporation of bacteria was also achieved by using metallodlectric Janus particles as electrodes [12].

### 1.2. Focal Adhesion

Focal adhesions (FAs), as the contact sites between the cell and extracellular matrix (ECM), are believed to play key roles in various biological processes [13]. For example, by serving as the hub for information exchange, FA allows cells to sense and respond to signals/stimuli from their micro-environment. In addition, FA is also believed to be heavily involved in directing the fate and function of cells like migration and force-mediated remodeling of cytoskeleton [14]. Structure-wise, the growth of FA requires the assembly of a large set of proteins like talin, α-actinin, vinculin, paxillin, etc., to form an adhesion plaque. One side of such plaque is anchored to the ECM through integrin (a transmembrane adhesion protein), while the other side is connected to the cytoskeleton via the so-called stress fibers (i.e., F-actin bundles and associated myosin motor proteins) [15]. Among different proteins mentioned above, vinculin has received great attention because of its critical role in regulating the formation and functioning of FAs. For instance, through the interaction with talin, vinculin is engaged to the cytoplasmic tail of β integrin, eventually connecting the cell to the outside [16]. When there is a lack of vinculin, fewer and smaller adhesions between the cell and ECM can be formed. Wound healing also becomes slower for vinculin-negative cells. Interestingly, vinculin-overexpressed cells were found to have larger FAs but reduced motility, indicating a negative correlation between vinculin expression and the migration capability of cells [17]. Besides vinculin, another protein heavily involved in cell adhesion is actin. Specifically, the contraction forces generated by actin stress fibers are known to be essential for the stability of FAs as well as the rearrangement of cell cytoskeleton [18].

In addition to facilitating the formation of FAs, the forces generated by F-actin (eventually transmitted to the ECM through vinculin-integrin-ECM connections) are also responsible for driving other processes such as cell spreading and migration [19,20]. These forces can come directly from actin polymerization (i.e., the elongation of F-actin) or arise from myosin contraction (causing two F-actin to slide with each other), depending on the specific phenomenon being examined.

### 1.3. Cell Motility

Cell motility is involved in numerous important physiologic and pathologic processes such as wound healing, embryo development and cancer metastasis [21,22]. Typically, for migration to take place, a cell needs to be polarized first. After that, the propelling force generated by the directional polymerization of F-actins can then drive the cell to move [23,24]. Specifically, the continuous pushing of polymerizing actin filaments on the cell membrane results in the formation of pseudopods at the leading edge of motile cells, which can then be stabilized by strong cell-ECM adhesion. After that, internal contractions generated within the cytoskeleton can disrupt the adhesions at the rear end of the cell and then drag the cell body forward [25,26]. Evidently, proper coordination/synchronization of actin dynamics and turnover of FAs is essential for the persistent movement of cells.

### 1.4. Membrane Resealing

When the integrity of its plasma membrane is disrupted, a eukaryotic cell has the amazing ability to repair the damage—a process called membrane resealing, which is critical to maintain cell homeostasis and prevent cell death [27]. However, research into this intriguing phenomenon is rather limited. As mentioned above, membrane pores can be created by an electric field applied to the cell. Usually, after the removal of a short-duration electrical pause, holes on the membrane can be self-resealed, which is known as reversible electroporation [28]. On the other hand, it has also been found that once the magnitude or duration of the applied electric field is beyond a threshold level, damage on the cell membrane can be permanent (i.e., membrane pores in this case can no longer be resealed, which eventually results in cell lysis), a phenomenon known as irreversible electroporation [28]. Interestingly, recent evidence has suggested that the resealing behavior of cells might be related to their physiological or pathological state. For example, it was found that the resealing velocity of tumor cells (after mechano-poration) appears to be proportional to their drug resistance [6], indicating the possibility of utilizing membrane resealing time (or speed) as a marker in cancer classification.

### 1.5. Electroporation Chip

To investigate the response of different cancer cells, we designed a micro-electroporation system in our previous study [6,7]. We showed that the electroporation efficiency of lung tumor cells is closely related to their resistivity against Erlotinib (a commonly used drug for treating lung cancer). Nevertheless, the possibility of utilizing electroporation in interrogating the resealing dynamics of a cell membrane as well as achieving live cell staining (and hence elucidating the role of different important proteins in various cellular processes) has not been carefully explored.

**Figure 1:**
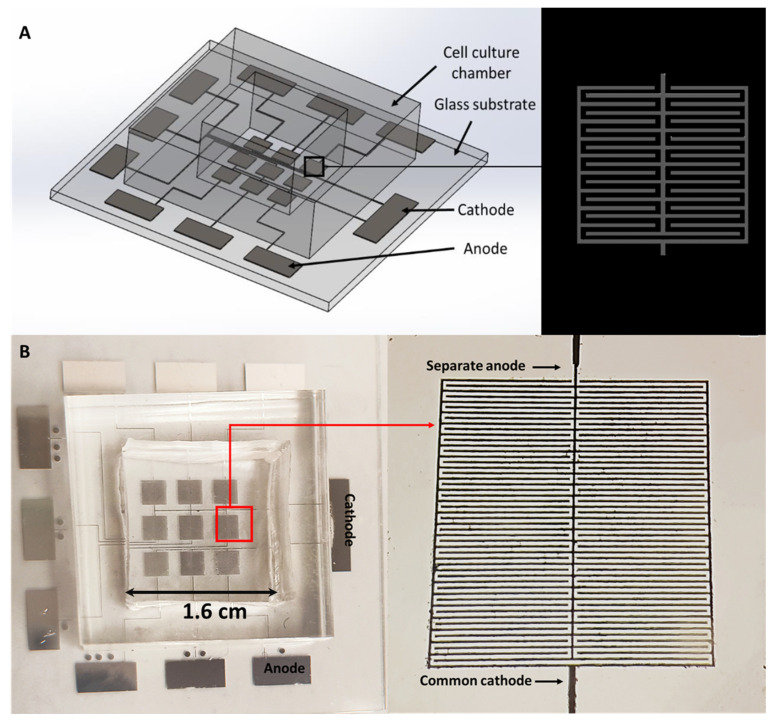
(**A**) Schematic plot showing the design of the micro-electroporation system. (**B**) Actual image of the fabricated chip containing nine electroporation regions. Each electroporation area has its own anode, while sharing a common cathode, as illustrated in the amplified image of one electroporation region, shown on the right.

In this study, a novel electroporation chip, containing nine independent electroporation regions, was developed, where each electroporation area has its own anode while sharing a common cathode (Figure 1). In this way, the same batch of cells cultured on the chip can be electroporated in a spatially and temporally controlled manner, allowing us to achieve fast staining of different targets inside the cell as well as systematically examining the resealing dynamics of the membrane. Specifically, we showed that electroporation-induced membrane pores in some cells could be resealed in ~10 min with our electric parameters, while irreversible electroporation can take place in other cells. In addition, GFP-vinculin plasmid (a protein heavily involved in the formation of cell adhesion) and 543 phalloidin (labeling filamentous actin) were successively delivered into the cell, allowing us to examine the individual and interactive dynamics of vinculin and F-actin during processes like cell adhesion and migration.

## 2. Material and Method

### 2.1. Fabrication of the Microelectrodes on a Glass Substrate

Aluminum (Al) microelectrodes were fabricated on glass in the present study. Specifically, a five μm Al layer was sputtered on a glass wafer. After that, the Al was coated with a photoresist layer by spin coating (SVG 8600 Series Track, Silicon Valley Group, CA, USA, at 4000 rpm for 30 s). To fabricate the designed geometry, we used the photolithography method (Karl Suss MA6 Mask Aligner, SUSS MicroTec Group, Garching, Germany) to introduce the pattern on the substrate with a bright field writing mask. Finally, the exposed aluminum was removed by dry etching (AST Cirie 200 Etcher, Advanced System Technology, Taiwan, China), and the photoresist was then stripped off from the substrate by plasma heating (Tepla 210 microwave O2 plasma system, PVA TePla AG, Wettenberg, Germany). Note that in the current design we do not have control over the voltage applied to cells outside the electrode region (or plane). Instead, our focus was on whether fast transfection, live cell staining and resealing dynamics monitoring can be achieved on cells attached/sitting on the electrode.

### 2.2. Program-Controlled Pulse Generator

The magnitude of the voltage supplied to the electrodes was regulated by a variable DC power supply. Other pulse parameters, such as the pulse duration and the pulse-to-pulse interval, were monitored by the Arduino processor controlled by the computer program. Two 10 kiloohm resistors and an NPN transistor formed the switch circuit, which is capable of achieving an ultra high-frequency switch control of the power supply, and hence can generate micro- to millisecond pulses.

### 2.3. Cell Culture

An NIH/3T3 fibroblast (American Type Culture Collection, Rockville, MD, USA) was used in the present study. Specifically, NIH/3T3 cells were cultured in DMEM (Dulbecco’s Modified Eagle Medium, Gibco, Life Technologies Corporation, Grand Island, NY, USA), supplemented with 10% fetal bovine serum (FBS) (GeminiBio, West Sacramento, CA, USA) and 1% penicillin–streptomycin (P.S.) (Gibco, Life Technologies Corporation, Grand Island, NY, USA), and incubated at 37 °C in a humidified atmosphere containing 5% CO_2_. Cells were plated on the electroporation chip coated with fibronectin for 12 h before electroporation. After electroporation, cells were cultured in the same medium.

### 2.4. Plasmid Extraction

GFP-mouse vinculin full length (889), a gift from Alpha Yap (Addgene plasmid # 67935; http://n2t.net/addgene:67935; RRID: Addgene_67935), was used for electrotransfection in this study. DNA isolation was achieved with plasmid purification kits (PureLink™ HiPure Plasmid Kits, Thermo, Waltham, MA, USA).

## 3. Results and Discussion

### 3.1. Characterizing the Resealing of Membrane Pores Induced by Electroporation

**Figure 2:**
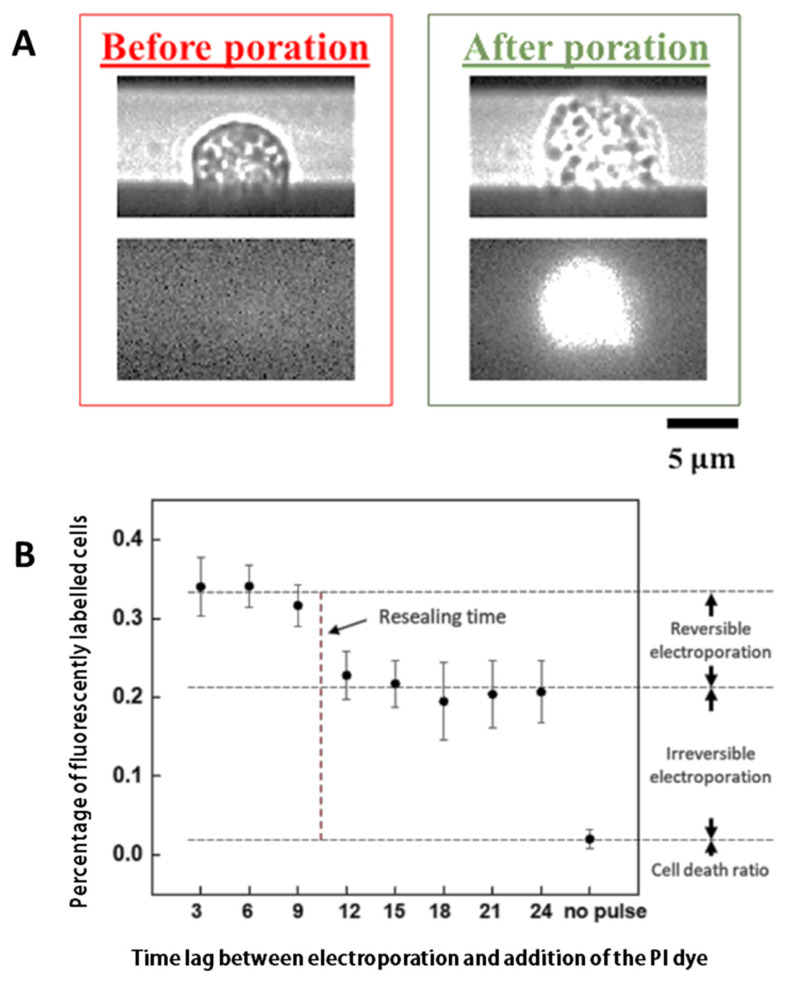
(**A**) Representative phase contrast (upper) and fluorescent (lower) images of cells before and after electroporation. (**B**) Percentage of fluorescently labeled NIH/3T3 cells as a function of the time between electroporation and the addition of the propidium iodide (PI) dye. Measurement was conducted on ~1000 cells in the nine electroporation regions, and data were presented as mean ± S.D (n = 5). The rightmost data point shows that a tiny portion of cells died during the experiment, allowing PI dyes to enter them even without electroporation (as a control test).

NIH/3T3 Cells cultured in nine regions were electroporated with the same electric parameters (three 10 V electrical pulses with a duration of 0.5 ms and a pulse-to-pulse interval of 1 s) in sequence with an interval of 3 min between each region. After electroporation was introduced to all nine areas, propidium iodide (PI) dyes were added to the medium and then the portion of fluorescently labeled cells in each region was calculated. Note that in this way dyes were added to cells in the first and eighth regions 24 and 3 min after electroporation, respectively. The percentage of fluorescently labeled cells as a function of the time lag between electroporation and addition of PI dyes is shown in Figure 2. Interestingly, a significant drop (from ~30% to 20%) in the portion of fluorescent cells was observed between 9 and 12 min, indicating that membrane pores in some electroporated cells have been resealed (i.e., the electroporation on these cells was reversible). In contrast, even when dyes were added 24 min after electroporation, they could still enter ~20% of cells, suggesting that their electroporation is irreversible, that is, permanent pores/damages have been introduced to the membrane of these cells.

### 3.2. Achieving Live Cell F-Actin Staining

**Figure 3:**
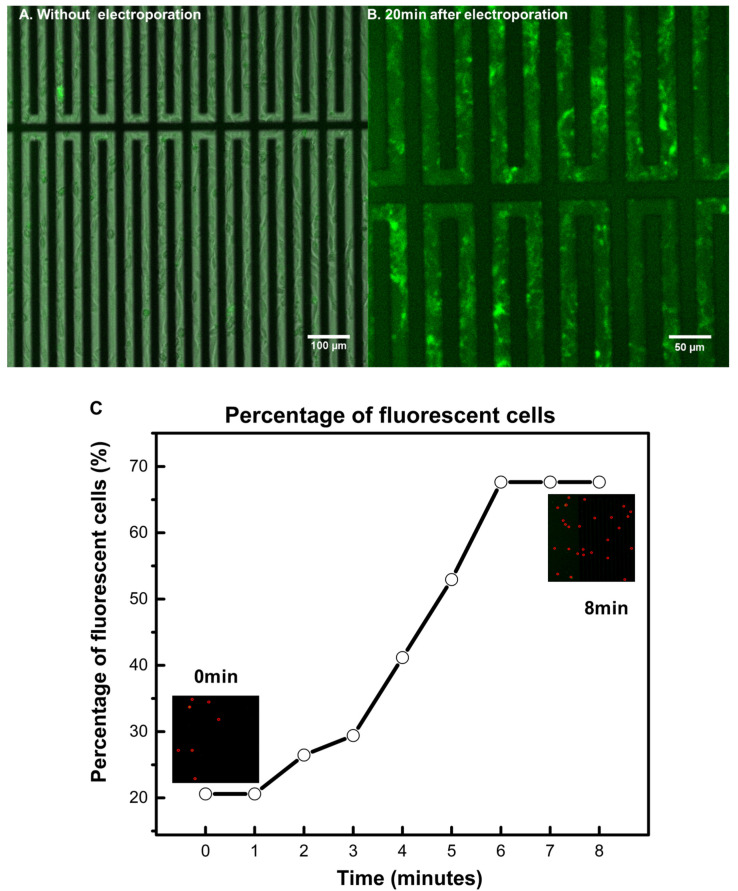
(**A**) Representative fluorescent image of NIH/3T3 cells without electroporation. The few fluorescent spots likely came from apoptotic cells. (**B**) Representative fluorescent image of cells 20 min after electroporation. Clear staining of F-actin (by phalloidin) in many cells can be observed. (**C**) The percentage of fluorescently labelled cells (marked by the red dots in the insets) as a function of time after electroporation. The percentage was found to quickly rise to a constant level in the first few minutes (after electroporation) and then more or less stayed at that level afterward. Note that, because the chip had to be moved under a microscope after electroporation, 0 min here actually does not mean immediately after electroporation. (Confocal laser scanning microscope: Carl Zeiss LSM 710 NLO, Jena, Germany).

To demonstrate the live cell staining capability of the electroporation system, Alexa Fluor™ 488 phalloidin (a high-affinity F-actin probe) was added to the culture medium of NIH/3T3 cells. After that, cells in the nine regions were electroporated with the same electric parameters (four 15 V electrical pulses with a duration of 0.5 ms and a pulse-to-pulse interval of 1 s) in sequence with no time delay between them. To detect a clear fluorescent signal from the cells, the medium containing fluorescent dyes was replaced after electroporation. Furthermore, the chip was also gently rinsed with PBS to remove possible deposited dyes. Interestingly, after electroporation, clear fluorescent signals were observed in many cells (Figure 3A,B), proving that live cell staining has indeed been achieved. Moreover, as illustrated in Video S1, the percentage of fluorescently labelled cells actually varied with respect to time after electroporation. Specifically, as shown in Figure 3C, the percentage was found to increase quickly to a “steady-state” value within a few minutes of electroporation and then more or less remained at that level after that. We believe this demonstrates the gradual deposition/binding of phalloidin to F-actin in cells and then the saturation of the number of cells labelled by such dye. Notice that the chip had to be moved under a microscope after electroporation, so 0 min in Figure 3C does not mean immediately after electroporation. Another point to make is that, interestingly, Figure 3C also shows that the percentage of fluorescently labelled adherent cells stopped to increase after ~6+ min of electroporation, which is comparable to the resealing time (~10 min) of membrane pores in floating cells discussed earlier (Figure 2B).

### 3.3. Achieving Fast Electrotransfection

**Figure 4:**
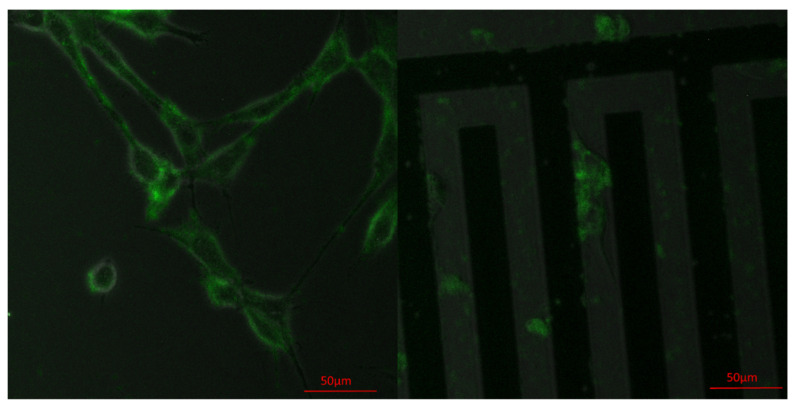
Representative confocal (Carl Zeiss LSM 710 NLO) images of NIH/3T3 cells, 24 h after electrotransfection, in (**Right**) and away from (**Left**) the electrode region.

Fast electrotransfection can also be achieved on our system. For example, after adding GFP-mouse vinculin plasmid (with a final concentration of 25 ng/μL) to the culture medium, we have electroporated NIH/3T3 cells with four 15 V electric pulses, with a duration of 0.5 ms and a pulse-to-pulse interval of 1 s. Figure 3 shows the fluorescent image of cells after electroporation. Evidently, vinculin has been labeled successfully inside the cell. This result indicated that our micro-electroporation chip could be useful in real-time monitoring of different cellular processes. One thing worth noting is that, in principle, only cells near the electrodes will be electroporated, so the fluorescent signal should localize in the electrode region. However, 24 h after electroporation, fluorescence was also observed in cells away from the electrodes (Figure 4), suggesting that many cells have migrated out from the electrode region.

### 3.4. Vinculin Dynamics during Cell Migration

**Figure 5:**
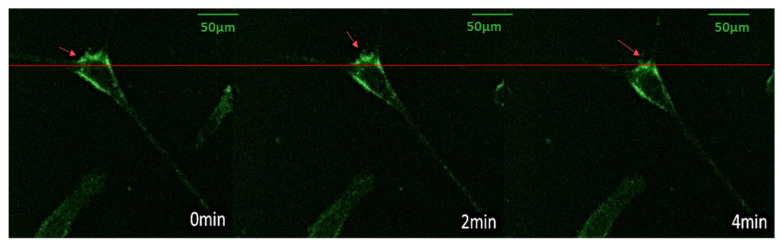
GFP-vinculin fluorescence images of a migrating NIH/3T3 fibroblast at different time points. The arrow indicates the moving (as well as the pseudopodia contraction) direction of the cell.

As mentioned above, NIH/3T3 cells appeared to maintain migration capability after electrotransfection. Therefore, we proceeded to monitor the dynamics of vinculins (a protein known to be heavily involved in the formation of FAs) inside the cell during its migration. As illustrated in Figure 5 as well as in Video S2, significant morphological changes of cells took place during their locomotion. Specifically, as the cell slowly moved forward, some pseudopodia dragged behind (presumably due to strong cell–substrate adhesion, as suggested by the vinculin signals there), which then retracted intermittently towards the cell body from time to time. These results demonstrate the potential of using the micro-electroporation setup developed here for real-time monitoring of different cellular processes as well as interrogating the role of key molecules involved.

### 3.5. Successive Labelling of Vinculin and F-Actin

**Figure 6:**
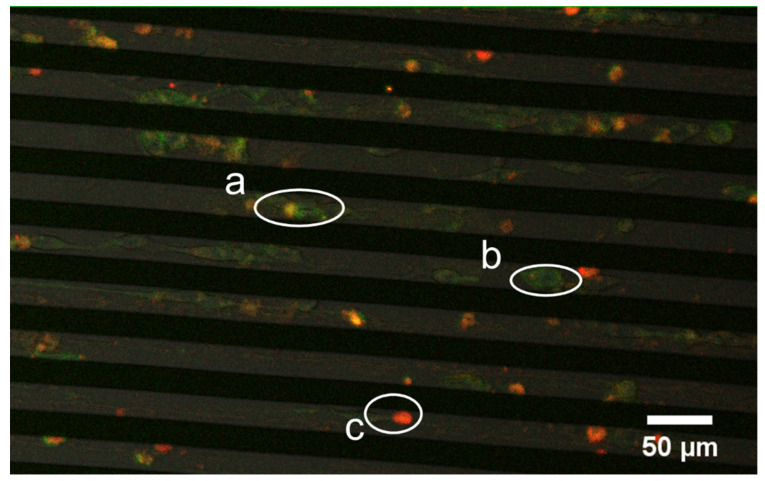
GFP-vinculin (green) and an F-actin fluorescence image of NIH/3T3 fibroblasts: a—cell with successful phalloidin-actin binding (orange); b—cell without phalloidin-actin binding; and c—an apoptotic cell (phalloidin appears in red without F-actin binding).

Given that most cellular processes, such as cell migration and adhesion, require precise coordination among various proteins, elucidating the interplay or interaction between these molecules could be critical for our understanding of how cells perform different functions. In this regard, our system could serve as a useful tool in achieving this. For example, after transfecting NIH/3T3 cells with GFP-vinculin plasmid, we further delivered phalloidin into these cells with our electroporation chip (following the same protocols mentioned above). Consequently, both vinculin and F-actin were labelled inside the cell. The presence of vinculin is represented by green fluoresces in the confocal image shown in Figure 6 whereas the red fluoresce corresponds to phalloidin, which then turned into orange after successfully binding with F-actin. The actin and vinculin dynamics inside a cell during its morphological change or movement (refer to Video S3) can then be closely examined. These results demonstrate the capability of our micro-electroporation setup in delivering different dyes or particles into the cell and then closely monitoring their individual as well as collective roles in different processes in real-time.

## 4. Conclusions

In this study, we developed a novel electroporation system to introduce nano-sized membrane pores to cells in a spatially and temporally controlled manner. Using this setup, it was found that both reversible and irreversible electroporation can be triggered in the cell population, with transient membrane pores in the former case being able to reseal in ~10 min. Furthermore, GFP-mouse vinculin plasmid has also been successfully delivered into fibroblasts, enabling us to monitor in real-time the role of such adhesion proteins in the subsequent migration of cells. Interestingly, after electrotransfection of GFP-vinculin plasmid, we further delivered phalloidin (a fluorescent F-actin probe) into the fibroblasts, demonstrating the capability of our setup in delivering multiple dyes/particles into live cells in a controllable manner, which can greatly help researchers in elucidating the individual and collective roles of different proteins in various cellular processes. We also want to point out that, compared to conventional methods such as bulk electroporation [29] (requiring high-operation voltage, typically of the order of hundreds of volts) and rather expensive transfection reagents like lipofectamine [30], our micro-electroporation approach could offer advantages such as easy/safe to operate, low cost and real-time monitoring.

Given that the nine electroporation regions in our chip can be controlled separately, this setup might be useful in identifying desirable electroporation protocols (in terms of, for example, the amplitude, duration and number of pulses to be applied) for specific transfection or drug delivery purposes. In addition, the nine independent electroporation regions also provide an ideal platform for us to carry out precisely controlled comparison experiments on the same batch of cells. For example, different dyes, drugs, and inhibitors (targeting proteins of key interest) can be delivered to cells at different regions and at different time points. After that, the influence of these molecules/chemicals on functions such as cell spreading, division, and migration can then be quantitatively monitored and compared in real-time, a feature that could be very useful in future biomedical/biophysical studies.

## Supplementary Materials

The following are available online at https://www.mdpi.com/2072-666X/11/8/767/s1: Video S1: Gradual F-actin labelling of cells after electroporation, Video S2: Vinculin dynamics in a motile NIH/3T3 cell, and Video S3: F-actin labelling in cells with GFP-vinculin expression.
